# Synchronous Colon Adenocarcinoma and Renal Cell Carcinoma: Diagnostic Challenges and Simultaneous Laparoscopic Management in Two Cases

**DOI:** 10.3390/diagnostics16020287

**Published:** 2026-01-16

**Authors:** Cristian Iorga, Cristina Raluca Iorga, Victor Strambu

**Affiliations:** 1 Faculty of Medicine, “Carol Davila” University of Medicine and Pharmacy, 050474 Bucharest, Romania; cris.iorga@yahoo.com (C.I.); dr.strambu@gmail.com (V.S.); 2 Surgery Clinic, “Dr. Carol Davila” Clinical Nephrology Hospital, 010731 Bucharest, Romania

**Keywords:** synchronous tumors, colon adenocarcinoma, renal cell carcinoma, laparoscopic approach, diagnostics and oncological tactics

## Abstract

**Background:** There is an increasing number of synchronous tumor diagnoses, mainly due to new investigative techniques and diagnostic guidelines. While renal and colonic malignancies are common, synchronous cases remain rare. They are usually diagnosed during the staging work-up performed for the primary cancer. **Case Presentation:** We share our experience with two cases of synchronous colon adenocarcinoma and renal cell carcinoma. The surgical intervention was performed simultaneously and laparoscopically, with good results and prognosis. Reviewing the literature, we found few studies reporting these synchronous tumors, which reflects their low incidence. Renal tumors are often identified during imaging studies performed for staging colonic tumors, and performing surgical treatment during the same operation is widely accepted. We performed a search of the literature to identify similar cases and to look for associations that can lead to synchronous colonic and renal malignancies. We also wanted to highlight the potential for therapeutic management as a single step, thereby avoiding a second surgical procedure. **Conclusions:** Synchronous renal and colonic malignancies are rare and are generally sporadic. Due to their rarity, there are no established guidelines, and management can be challenging. Presently, the treatment needs to be individualized based on discussions from the tumor board.

## 1. Introduction

Multiple primary malignant tumors (MPMTs) are no longer surprising, although diagnosing them remains challenging. Their incidence ranges from 0.73% to 11.70% [1]. Reimer and Billroth first described the concept of MPMTs in 1889, and Waren et al. established the criteria for diagnosing synchronous MPMTs (MPMTS), namely two or more tumors that are not metastatic from each other with different locations and histopathological features, diagnosed within a maximum of 6 months after the first tumor is identified [2,3]. The incidence of synchronous colon and kidney tumors varies between 0.4% and 4.85% [4,5].

MPMTS in renal tumors is rare, and it is most often associated with other urinary tract tumors (prostate, bladder) and, to a lesser extent, with colorectal, lung, or malignant melanoma tumors [6]. Beisland et al.’s study reports an overall MPMTS incidence of 16.1% in kidney tumors [6]. MPMTS in colon tumors is more frequently associated with lung, breast, genital, and renal tumors [7]. Meanwhile, the overall occurrence of MPMTS in colon tumors is 5% according to Cochetti et al. [8].

CRC is the third most prevalent malignancy worldwide, with an incidence of 8%, and is a major contributor to cancer-related mortality, while RCC accounts for 2–3% of cancer cases in adults [1,9].

Male dominance is often reported because both renal cell carcinoma (RCC) and colorectal cancer (CCR) have a higher incidence in men. While coincidence may explain many cases, several shared risk factors support a biological relationship, including smoking, obesity, metabolic syndrome, chronic inflammation, oxidative stress, high-fat and low-fiber diets, and genetic syndromes like Lynch and Von Hippel–Lindau syndrome [4,9].

Hypotheses for molecular mechanisms include DNA mismatch repair defects, oncogenic mutations caused by microenvironmental dysregulation and chronic inflammation, and overlapping angiogenic pathways (associated with aggressive tumors) [9].

Detecting synchronous renal masses during CRC staging has become increasingly common due to the routine use of contrast-enhanced computed tomography (CT). Most incidental renal lesions identified in this context are benign; however, up to 1–3% are subsequently diagnosed as RCC. Population-based studies suggest a modestly increased lifetime risk of developing secondary primary malignancies in RCC survivors, including CRC, which is potentially linked to common etiologic factors [9].

The surgical management of these complex MPMTS cases presents new challenges in addition to multimodal oncological treatment. Initially, surgical treatment only targeted the tumor with the worst prognosis and was performed solely by open methods. At present, owing to technological progress, surgical interventions can be performed with minimal invasion—laparoscopically or robotically—and can address both tumors in the same procedure [1,4,7,8,9,10].

Subsequent oncological treatment is administered in CRC according to TNM staging, whereas RCC is generally chemo-resistant and involves targeted therapies (tyrosine kinase inhibitors and immunotherapy). Importantly, CRC chemotherapy does not adversely affect RCC biology [7,8].

Prognosis in synchronous CRC–RCC mainly depends on the stage of each tumor individually, with no evidence that simultaneous occurrence inherently worsens outcomes. In localized RCC (T1–T2), there is excellent survival (5-year >90%), while CRC survival depends on TNM staging, lymph node involvement, and MSI status. The incidental detection of early RCC during CRC staging may even improve survival [7].

This study was prompted by two cases of synchronous colon and kidney tumors encountered in our clinical practice. We aimed to identify a possible relationship between the two types of tumors to assess their incidence, diagnostic approaches, and treatment options for these complex cases, and reviewed the literature based on our experience.

## 2. Materials and Methods

The Surgery Clinic at Dr. Carol Davila Clinical Hospital treats malignant diseases—including digestive, urological, and genital cancers. In our experience, we have encountered patients with synchronous tumors—such as those in the colon and kidney, breast and colon, or rectum and retroperitoneum. Managing these patients has always been challenging, as there are no established guidelines for a preferred treatment sequence. However, during tumor board meetings, we have decided that, in selected cases, surgery will be performed in the same operative session with a combined team (general surgeon and urologist). Below, we share our experience with two cases of synchronous colon and kidney tumors. The procedures were performed by the same surgical team under general anesthesia. Our approach was to first carry out a laparoscopic partial renal resection, followed by a laparoscopic colonic resection. We include details about the duration and outcomes, even in cases that initially seemed difficult.

We also conducted a scoping literature review in the following databases: PubMed, PubMed Central, Google Scholar, and Cochrane to search for clinical cases or series of synchronous colon and renal cancer. The search yielded 6590 articles. The study was conducted between 15 November 2024 and 15 February 2025 and received approval from the local ethics committee.

The inclusion criteria were as follows: articles in English; patients over 18 years old; articles describing synchronous colon and kidney tumors; and case reports or series. Only 33 articles meeting these criteria were identified [4,5,6,7,8,10,11,12,13,14,15,16,17,18,19,20,21,22,23,24,25,26,27,28,29,30,31,32,33,34,35,36,37].

## 3. Case Presentation

### 3.1. Case 1

**Figure 1:**
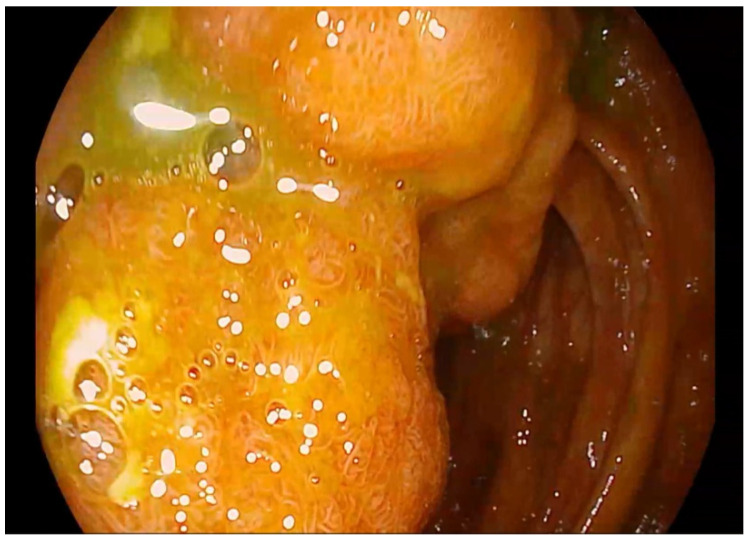
Colonoscopic view of the giant cecal polyp.

A 64-year-old male patient was referred to the General Surgery Clinic at Dr. Carol Davila Nephrology Clinical Hospital in December 2024 to evaluate a large cecal polyp and determine the appropriate treatment. The patient had no known family or personal medical history, was a non-smoker, and had a BMI of 24. Clinical presentation included no specific complaints, pale skin, stable hemodynamic and respiratory status, a soft abdomen with no pain, normal bowel movements, and no changes in stool. The cecal polyp was diagnosed via colonoscopy two weeks prior to admission, when the patient was evaluated for anemia, with Hb 9 g/dL (Figure 1).

**Figure 2:**
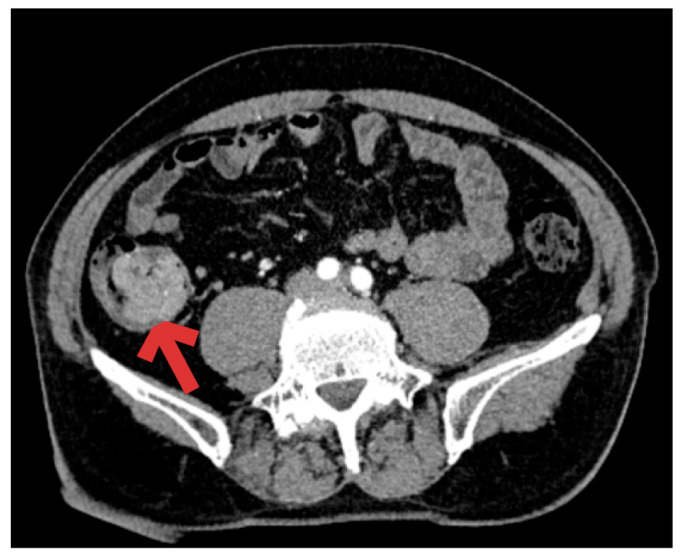
CT image—arrow indicates the cecal tumor.

The polyp measured 6/5/3 cm. During the colonoscopic procedure, a biopsy was taken, which revealed an advanced villous adenoma with low-grade epithelial dysplasia. Due to the polyp’s large size and the technical difficulty of obtaining a biopsy from its base, there was a high suspicion of malignant transformation. Accordingly, TNM staging investigations were performed in case the final histopathological examination indicated malignancy (Figure 2).

Tumor markers CA 19-9 and the carcinoembryonic antigen were within normal limits.

**Figure 3:**
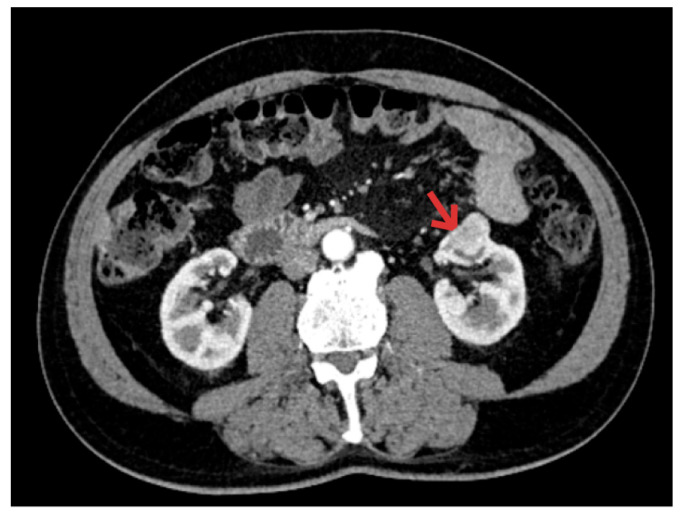
CT image—arrow indicates the renal tumor.

What was surprising was the CT scan of the abdomen, which revealed, in addition to the cecal tumor, a 2.5 cm superior left polar renal tumor consistent with clear cell renal carcinoma and no detection of adenopathy (Figure 3).

Given the strong suspicion of renal tumor malignancy, a tumor board was convened to determine a treatment plan. Possible treatment options were discussed, taking into account the polyp’s size and the lack of a histopathological confirmation of malignancy. The decision was difficult, but we had to consider the complexity of colonoscopic resection and the risks of incomplete treatment.

Finally, the therapeutic option included performing radical surgery for both tumors, and depending on the final anatomopathological diagnosis, the patient would be referred to the oncology department. In the same operative procedure, a laparoscopic partial resection of the upper left polar renal pole and right hemicolectomy with ileo-transverse mechanical termino-lateral anastomosis was performed. The surgery lasted 268.8 min, and the partial renal resection took 141 min. The definitive histopathological result showed that the renal tumor was clear cell carcinoma, G3, without lymphovascular invasion, staged as pT1aN0M0. The cecal polyp was identified as an advanced villous adenoma with low-grade epithelial dysplasia and foci of adenocarcinoma in situ without evidence of microsatellite instability in immunohistochemical tests. The patient’s postoperative course was favorable, and he was referred to the oncology department for further care management. The team decided not to initiate treatment but to monitor the patient annually through oncological surveillance. This case represents the second instance of synchronous kidney and colon tumors that we have encountered in our practice over the past 27 years.

### 3.2. Case 2

A 54-year-old female patient arrived in June 2014 for tests due to rectal bleeding that began two months prior. Her family history included breast cancer in her mother and cardiovascular issues. She was known to have high blood pressure and dyslipidemia and was taking amlodipine and atorvastatin. Clinical examination showed a patient in good overall condition, with normal skin color, and she was hemodynamically and respiratorily stable. The abdomen was distended due to adipose tissue, and the patient had a BMI of 28.65. Rectal examination revealed no abnormalities.

During colonoscopy, a tumor at the splenic flexure of the colon was observed, nearly completely occluding the colonic lumen and preventing passage during the procedure. A biopsy confirmed G2 colonic adenocarcinoma. The subsequent TNM staging unexpectedly revealed a right upper pole renal tumor consistent with clear cell renal carcinoma, in addition to the colon tumor, with no significant lymphadenopathy detected. Tumor markers CA 19-9 and carcinoembryonic antigen levels were within normal limits, as were the other laboratory tests, except for hemoglobin, which measured 9.5 g/dL. To determine the treatment plan, a tumor board was convened, and we decided to perform a primary radical surgical resection of both tumors, followed by oncologic treatment based on the final pathological results. The procedure was performed via a laparoscopic approach, including a right renal partial resection and a left hemicolectomy with mechanical side-to-side colo-colic anastomosis. The surgery lasted 150 min, with the partial renal resection taking 65 min. The final histopathology report showed a renal tumor with clear cells and a G2 colonic adenocarcinoma, without vascular or perineural invasion. However, there was an invasion of 2 out of 24 examined lymph nodes (staging pT2N1M0). Immunohistochemistry tests revealed no evidence of microsatellite instability and a Ki index of 15%. The postoperative course was complicated by the development of an anastomotic fistula that did not respond to conservative treatment, necessitating reintervention and the creation of a temporary colostomy on the transverse colon. Subsequently, the patient was referred to the oncology department and underwent oncologic treatment (FOLFOX) for six months. Twenty-four months after surgery, regular check-ups indicated that the oncological disease was stable (tumor markers within normal limits and no signs of local recurrence or distant metastases on CT scans and colonoscopy). Therefore, it was decided to reintegrate the colostomy into the digestive system.

## 4. Discussion

The vast majority of published studies report one or at most two cases encountered in the surgical activities of their respective teams [10,11,12,13,15,16,17,18,19,20,21,22,23,24,33,34,35]. Cases with five or six consecutive instances are rare, highlighting the low incidence of synchronous colon and kidney tumors [13,14].

Analyzing the selected studies reveals two common themes among the authors, as follows:

- The second synchronous tumor is usually a renal tumor, found during additional preoperative tests for colon tumor staging. This underscores the value of imaging not only for metastatic assessment but also for identifying additional primary malignancies. Our study confirms this frequent finding in the literature.
- We did not find any studies where the first diagnosed tumor was renal, followed by a colonic tumor.

The clinical presentation is dominated by symptoms associated with CRC, including rectal bleeding, anemia, altered bowel habits, or obstructive features. In contrast, RCC is known as a “silent tumor”, with the classical triad—hematuria, flank pain, and a palpable mass—present in less than 10% of patients. Half of renal cell carcinoma cases are diagnosed incidentally for work-up performed for other conditions [9,13,14,15].

Regarding the cause of this disease, most studies emphasize common factors such as alcohol consumption, smoking, hormonal factors, and previous treatments like radiotherapy or chemotherapy [6]. Several studies explore the possible causes of synchronous colon and kidney tumors in Lynch syndrome I and II and microsatellite instability, especially MSI-H (microsatellite instability high) (gene mutations MLH1, MSH2, MSH6, and PMS2) [27,28,29,30].

In the presented cases, we did not detect Lynch syndrome or microsatellite instability. Based on published research and personal experience, no definitive conclusion can be made about the cause of colon+kidney MPMTS; it is very likely that the occurrence of the two tumor types is sporadic [30].

Imaging diagnostic methods (CT and MRI) are decisive in diagnosis and staging.

In recent years, some authors have suggested using positron emission tomography/computed tomography (PET CT) for detecting MPMTS. This method is especially useful when the first tumor identified is renal, as the reliability of CT in detecting colon tumors is low [31,32].

Surgery remains the definitive treatment for both CRC and localized RCC. Decisions regarding timing (combined vs. staged) must consider tumor size and complexity, the presence of obstruction or bleeding, patient comorbidities, and the surgeon’s expertise in minimally invasive combined procedures [12,15,16,17,18,19,20,21,22].

Combined resections are increasingly performed in cases of early-stage, uncomplicated tumors, offering reduced hospitalization and anesthesia exposure. Conversely, staged surgery is preferred when RCC is large or involves the renal hilum, CRC presents emergently, or when prolonged operative times may increase morbidity [15,17,21,22].

Most authors recommend performing surgery for both tumors at the same time, and increasingly, more authors support laparoscopic or recent robotic surgical techniques [6,7,8,9,10,11,12,13,14,15,16,17,18,19,20,21,22,23,24,25].

In recent years, an increasing number of studies have highlighted the possibility of performing concomitant laparoscopic surgery.

Although laparoscopic surgery typically takes about 100 min longer than open surgery—especially for right colon or left kidney procedures—it is preferred due to significantly faster postoperative recovery and lower postoperative analgesia requirements [8,12,13,15,16,20,21,23,24,25,33,34].

In terms of postoperative complications, recent studies show that they are similar between laparoscopic and open approaches, which supports the use of laparoscopic surgery despite the longer duration compared to open surgery [12,13,16,21,23,24,25,26,38].

In one of the presented cases, a postoperative anastomotic fistula occurred, but we were unable to link this incident to the type of surgery or to the prolonged duration of the concomitant surgical intervention.

Postoperative oncological treatment and prognosis for patients with MPMTS are topics that are seldom covered in the studies included in the review.

From a treatment perspective, most studies focus on discussing surgical options (laparoscopic or traditional surgical techniques and, more recently, robotics and especially surgical strategy), but very few studies also address postoperative oncologic treatment [1,8,10,11,12,13,14,15,16,18,19,20,21,22,34,35,39,40].

From a histopathological perspective, CRCs are adenocarcinomas, while RCCs are clear cell, papillary, or chromophobe carcinomas (listed in order of decreasing frequency). Independent origins must be confirmed histologically: CRCs are typically CDX2+, CK20+, or CK7–, and RCCs are usually PAX8+ or CAIX+ [39,40,41].

Histopathological evaluation rules out rare CRC metastasis to the kidney or vice versa.

As previously discussed, the most commonly detected tumor is colorectal, which is typically in the most advanced stage. The oncological treatment recommended in the studied research is most commonly used for colorectal tumors according to the TNM stage, with no treatment for renal tumors, as shown in our case of synchronous colon and renal tumors. The treatment decision should be made by the tumor board to ensure the patient receives the most effective personalized care. Regarding prognosis (in articles where this is mentioned), it is considered to be one of the most advanced oncological tumors and not viewed as having a worse prognosis due to the presence of both tumor types [36,37,41,42].

In terms of postoperative therapeutic approaches, no consistent conclusions can be drawn that could serve as the basis for future guidelines for treating synchronous colon and kidney tumors.

In the absence of guidelines for synchronous MPMTs for both the diagnostic stage and especially for the surgical and oncologic treatment stage, it is very important that the therapeutic strategy be performed by a multidisciplinary team and the final decision be made by the tumor board [1,11,13,15,17,20,21,22,23,24,40,43,44].

As a novelty, this article attempts to systematize the diagnostic method, causes, prognosis, and surgical and oncological treatment modalities of synchronous colon and kidney tumors, drawing attention to the need to adopt current medical practice guidelines.

The study has several limitations, mainly due to its retrospective nature and the data collected from articles, most of which are case reports with a small number of patients. The data presented are unsystematic and inconsistent. We believe that more research is necessary to include standardized information on MPMTS tumor diagnosis, investigation methods, comprehensive anatomopathological diagnosis, and surgical and oncological treatment approaches so that specific guidelines can be established for these patients.

## 5. Conclusions

With advances in imaging, the diagnosis of synchronous MPMTs is increasing and can pose challenges in patient management.

A multidisciplinary approach ensures optimal management, with single-stage laparoscopic or robotic combined surgical resection increasingly recognized as safe and effective. The continued accumulation of clinical data is necessary to refine treatment strategies and understand the biological interactions between these concomitant malignancies.

## Data Availability

The original contributions presented in this study are included in the article. Further inquiries can be directed to the corresponding author.

## Abbreviations

The following abbreviations are used in this manuscript:

MPMTs	multiple primary malignant tumors
Hb	hemoglobin
TNM	tumor node metastasis
CA 19 9	carbohydrate antigen 19-9
CT	computed tomography
G3, G2	tumor grading 2 or 3
FOLFOX	chemotherapy with folinic acid, fluorouracil, and oxaliplatin
